# Stilbenes: a promising small molecule modulator for epigenetic regulation in human diseases

**DOI:** 10.3389/fphar.2023.1326682

**Published:** 2023-12-12

**Authors:** Jing Tian, Li Jin, Hongquan Liu, Zichun Hua

**Affiliations:** ^1^ The State Key Laboratory of Pharmaceutical Biotechnology, School of Life Sciences, Nanjing University, Nanjing, Jiangsu, China; ^2^ Jiangsu Province Hospital on Integration of Chinese and Western Medicine, Nanjing, China; ^3^ Changzhou High-Tech Research Institute of Nanjing University and Jiangsu TargetPharma Laboratories Inc., Changzhou, China; ^4^ Nanjing Generecom Biotechnology Co., Ltd., Nanjing, China

**Keywords:** stilbenes, epigenetic, Resveratrol, Pterostilbene, Tamoxifen, positive and negative regulation, small molecule modulator

## Abstract

Stilbenes are characterized by a vinyl group connecting two benzene rings to form the basic parent nucleus. Hydrogen atoms on different positions of the benzene rings can be substituted with hydroQxyl groups. These unique structural features confer anti-inflammatory, antibacterial, antiviral, antioxidant, anticancer, cardiovascular protective, and neuroprotective pharmacological effects upon these compounds. Numerous small molecule compounds have demonstrated these pharmacological activities in recent years, including Resveratrol, and Pterostilbene, etc. Tamoxifen and Raloxifene are FDA-approved commonly prescribed synthetic stilbene derivatives. The emphasis is on the potential of these small molecules and their structural derivatives as epigenetic regulators in various diseases. Stilbenes have been shown to modulate epigenetic marks, such as DNA methylation and histone modification, which can alter gene expression patterns and contribute to disease development. This review will discuss the mechanisms by which stilbenes regulate epigenetic marks in various diseases, as well as clinical trials, with a focus on the potential of small molecule and their derivatives such as Resveratrol, Pterostilbene, and Tamoxifen.

## 1 Introduction

Epigenetics is the study of heritable changes in gene expression or cell phenotype by mechanisms that do not alter the DNA sequence. Epigenetic phenomena include DNA methylation, RNA interference, and histone modification, among others (Goldberg et al., 2007). Serving as the bridge between genotype and phenotype, epigenetics plays a pivotal role in eukaryotic development, selectively regulating gene transcription (Yung and Julius, 2008; Tammen et al., 2013). The field of epigenetics has garnered significant attention and research due to its reversibility and plasticity. It has been found to play a regulatory role in various areas, such as cancer development, aging, cardiovascular and cerebrovascular diseases, as well as nervous system diseases. Consequently, several epigenetic modifiers have been identified as novel therapeutic targets, including DNA methyltransferases (DNMT), EZH2 methyltransferases, and Histone deacetylases (HDAC) (Lyko, 2018; Duan et al., 2020; Ramaiah et al., 2021).

Compounds can be classified into phenols, ketones, terpenoids, antibiotics, amino acids, and stilbenes. Among these, several compounds, in addition to stilbenes, are well-documented and extensively researched. Stilbenes, due to their unsaturated double bonds, exhibit robust reactivity and can undergo modification through addition and oxidation reactions, potentially resulting in compounds with substantial functional potential. Stilbenes are characterized by a core structure consisting of two benzene rings connected by a vinyl group, with hydrogen atoms in the two benzene rings replaced by hydroxyl groups to form polyols. Owing to the instability of their vinyl structure and susceptibility to isomerism, the majority of stilbenes exist in the trans configuration. These compounds and their derivatives have garnered significant attention in pharmaceutical research and development due to their potential applications in disease treatment and prevention. Examples include Resveratrol (RSV), Pterostilbene (PTE), and their dimers, such as (±)-trans-δ-vinifera (Giacomini et al., 2016; Kluska et al., 2023). They function as antioxidants and anticancer agents, modulating epigenetic marks that impact disease progression, particularly in terms of DNA methylation and histone modification (Ganguly et al., 2021; Levenson, 2022a; Sukocheva et al., 2022), which, in turn, can influence gene expression patterns and promote or hinder disease progression.

As an important avenue for regulating disease development, epigenetics has driven the discovery of small molecule drugs. A substantial body of research has confirmed that stilbenes, including Resveratrol and Pterostilbene, act as epigenetic modifiers to prevent cancer initiation and progression. Encouragingly, hundreds of clinical trials have been dedicated to the study of these stilbenes. How do stilbenes influence epigenetics to facilitate or suppress disease progression? This article reviews the interplay between the structure of stilbenes and epigenetics.

## 2 Structural and function of stilbenes and derivatives

**FIGURE 1:**
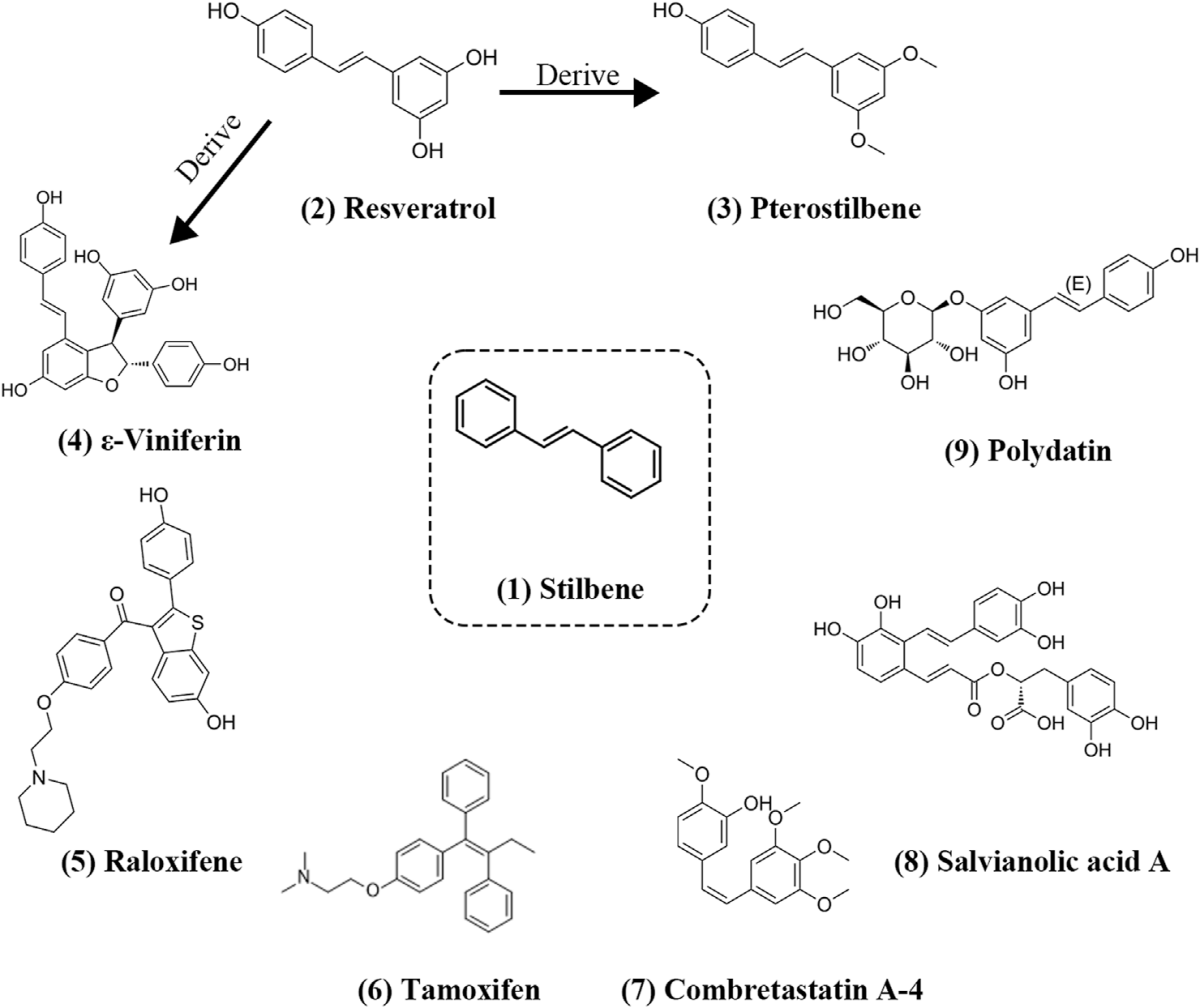
Structure of stilbenes involved in the regulation of epigenetic (Goldberg et al., 2007). Structure of the parent nucleus of stilbenes (Tammen et al., 2013); Structure of Resveratrol (Yung and Julius, 2008); Structure of Pterostilbene (Lyko, 2018); Structure of ε-Viniferin (Duan et al., 2020); Structure of Raloxifene (Ramaiah et al., 2021); Structure of Tamoxifen (Kluska et al., 2023); Structure of Combretastatin A-4 (Giacomini et al., 2016); Structure of Salvianolic acid A (Sukocheva et al., 2022); Structure of Polydatin.

Stilbenes exhibit remarkable versatility due to their stable xylene structure and the strong hydrogen bond substitution capability. Numerous stilbenes have been successfully identified, and structurally, they can be categorized as trans-stilbene and cis-stilbene (Giacomini et al., 2016). Both of these isomers are more stable in the trans configuration and can freely interconvert under UV light exposure (Piekuś-Słomka et al., 2019). The stilbene structure possesses several advantageous properties, including high thermal stability for photoisomerization and simplicity in structural synthesis (Giacomini et al., 2016; Villarón and Wezenberg, 2020). Notably, both Pterostilbene and ε-Viniferin are structural derivatives of Resveratrol, with Pterostilbene exhibiting superior antioxidant capacity. Among FDA-approved synthetic stilbene derivatives, Tamoxifen and Raloxifene are commonly prescribed. Trans-stilbene structures, exemplified by compounds like Resveratrol, Pterostilbene, and Tamoxifen, display greater structural stability than cis-stilbenes, as seen in the case of Combretastatin A-4 which is a trans-stilbene but structurally unstable (Paidakula et al., 2022),. Additionally, beyond cis and trans-stilbenes, there exist rigidly structured stilbenes such as Raloxifene. All these stilbenes play pivotal roles in mediating positive and negative feedback through epigenetic modifications in the context of tumor, metabolism, and neurological diseases. Information on some of the stilbenes that regulate epigenetic inheritance is shown in the Table 1 and their structure is shown in Figure 1.

**TABLE 1:** Main stilbenes regulate epigenetics.

Name	Structure	Pharmacological functions	Epigenetic target	Cite
Resveratrol	Goldberg et al. (2007)	Anti-aging, anticancer, anti-cancer, prevention of cardiovascular disease	Sirtuin1, DNMT3B, TET1-3, ESR1, stat3, HDACs, PterostilbeneN, MTA1, CDH263, CDKN1A, ATP2A3, Nrf2-Keap1 miR-539-5p	Phase 3
Pterostilbene	Tammen et al. (2013)	Anticancer, anti-inflammatory, antioxidant and analgesic agents	OCT1/DNMT3B, MTA1/HDAC1, fasn, HDAC2, has-miR-9/has-miR-2	Phase 3
ε-Viniferin	Yung and Julius (2008)	Inhibition of the CYP family, strong antioxidant activity	DNMT, irtuin1	Indication
Raloxifene	Lyko (2018)	Treatment of postmenopausal osteoporosis in women	LSD1/KDM1A, ESR1, HDAC	Market
Tamoxifen	Duan et al. (2020)	Treatment of recurrent or metastatic breast cancer	LncRNA DILA1, SALL2, ESR1, PterostilbeneN, miR-32-5p, HDAC, BARD1/BRCA1, MMP1	Market
Combretastatin A-4	Ramaiah et al. (2021)	Microtubule inhibitor that binds to β-tubulin	HDAC	Phase 3
Salvianolic acid A	Kluska et al. (2023)	Inhibits matrix MMP-9 and has anti-inflammatory effects to protect the blood-brain barrier	HDAC 10/STAT3, DNMT1, miR-152, Sirtuin 1	Phase 4
Polydatin	Giacomini et al. (2016)	Inhibit G6PD and induce oxidation and endoplasmic reticulum stress, anti-inflammatory	HDAC, Sirtuin 1, miRNA-21/miRNA-144-3p	Phase 2

## 3 Epigenetic regulation of stilbenes and their derivatives——positive regulation

### 3.1 Trans-stilbenes: Resveratrol and its derivatives

Resveratrol plays a significant role in orchestrating epigenetic modifications associated with various diseases, including cancer, metabolic disorders, cardiovascular conditions, and others, within the intricate landscape of disease inheritance (Singh et al., 2019). On a molecular level, Resveratrol exhibits a 3, 4′, 5-stilbestrol structure, which is recognized for its phytoantibiotic properties (Rauf et al., 2018). Resveratrol exists in both cis and trans configurations, with the primary focus of our study being directed towards its trans structure. The combination of glucose with these two structures yields cis and trans Resveratrol glycosides, respectively. When exposed to UV irradiation, trans-Resveratrol undergoes conversion into the cis-isomer. Resveratrol finds broad applications in the realms of food processing, skincare products, and pharmaceuticals (Ganesan and Choi, 2016; Pastor et al., 2019; Mirabelli et al., 2020; Ren et al., 2021). Clinical studies have unequivocally affirmed its lack of acute toxicity and genotoxicity, establishing it as a safe choice for human consumption and utilization (Movahed et al., 2020). Pharmacodynamic investigations have also shown that Resveratrol modulates epigenetic modifications associated with the onset and progression of diseases through the regulation of DNA methylation, histone acetylation, and microRNA (Table 2).

**TABLE 2:** Epigenetic regulation of different diseases by Resveratrol and its derivatives.

Name	Diseases type	Diseases	Regulated phenotypes	Regulatory mechanism	Stage
Resveratrol	Cancer	Melanoma	Inhibit cell migration	DNA methylation	Preclinical
		Thyroid cancer	Reverse retinoic acid tolerance	DNA methylation	Preclinical
		Glioblastoma	Cell cycle arrest and apoptosis	DNA methylation	Preclinical
		Colon cancer	Inhibit cell proliferation and invasion	Histone acetylation	Phase 1
		Ovarian cancer	Induces apoptosis	Histone acetylation	Phase 4
			Inhibit cell migration	MicroRNA Regulation	
		Prostatic cancer	Induces apoptosis	Histone acetylation	Preclinical
		Breast cancer	Induces apoptosis	Histone methylation and acetylation	Clinical
		Liver cancer	Increased expression of ZFP36	DNA methylation	Preclinical
	Metabolism	Non-alcoholic fatty liver disease	Diminish triglyceride accumulation	Histone acetylation	Phase 1
		Obesity	Inhibit *de novo* lipogenesis	MicroRNA	Phase 1
	Nervous	Parkinson	Attenuate MPP induced cell cycle arrest and cell apoptosis	DNA methylation and Histone acetylation	Phase 1
		Anxiety	Ameliorate hyper-anxiety associated with prediabetic state	DNA methylation	Phase 1
		Cognitive disability	Prevents cognitive decline in SAMP8 adult offspring	DNA methylation	Phase 3
ε-Viniferin	Cancer	Liver cancer	Reverse arsenic damage	DNA methylation	Preclinical
		Colon cancer	Reverse arsenic damage	DNA methylation	Preclinical
	Nervous	Parkinson	Inhibit apoptosis	Histone acetylation	Preclinical

#### 3.1.1 Resveratrol regulates cancer

##### 3.1.1.1 RSV and DNA methylation

**FIGURE 2:**
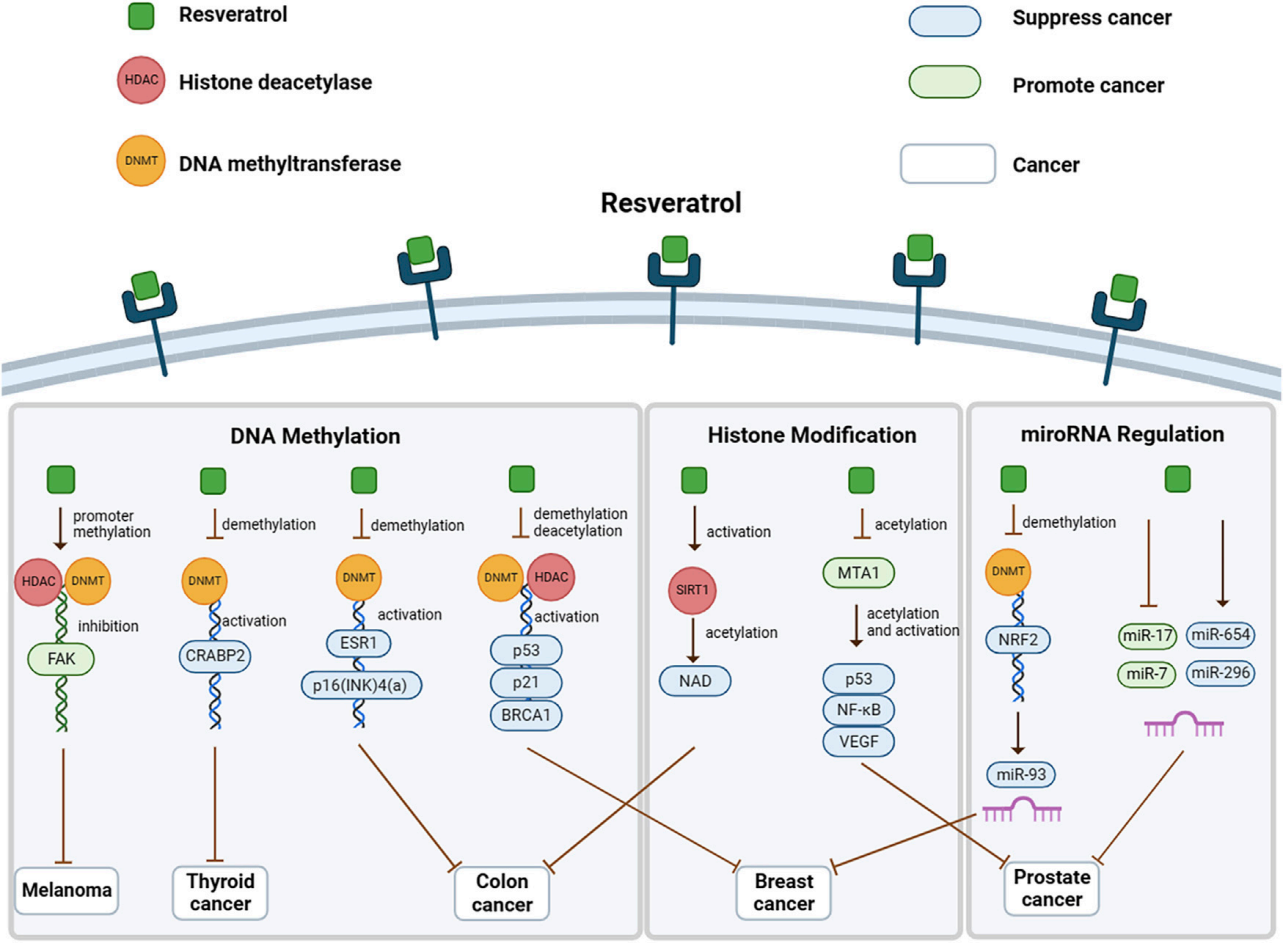
Mechanisms by which resveratrol regulates epigenetics in cancer.

Numerous studies have demonstrated the significance of Resveratrol in preventing cancer development. Resveratrol regulates the primary epigenetic alterations of tumors, including DNA methylation, histone acetyQlation, and modification of microRNA (Figure 2). In cancer cells, DNA methylation levels across genes are reduced, increasing mutation rates and impairing genomic stability (Johnstone et al., 2022; Sun et al., 2022). Conversely, through the abnormal activation of the expression of multiple proto-oncogenes, Resveratrol contributes to malignancy. The methylation level of CpG islands in certain genes is elevated, leading to an indirect induction of malignancy by decreasing the transcriptional activity of tumor suppressor genes. This subsequently affects gene expression, resulting in reduced expression or silencing of gene expression.

In melanoma, Resveratrol hinders cell migration by reducing the expression of focal adhesion kinase (FAK). Enhancing FAK expression boosts cell motility, while suppressing FAK signaling prevents cell migration (Mitra et al., 2005). Resveratrol brought HDAC1 and DNMT3a to the FAK promoter region, increasing promoter methylation and suppressing FAK expression, subsequently restraining melanocyte migration (Chen et al., 2018). In thyroid cancer, Resveratrol has been shown to cause the demethylation of the CRABP2 gene, which leads to increased tolerance of retinoic acid in cancer cells. Furthermore, Resveratrol can downregulate DNMT1, DNMT3A, and DNMT3B in cells, reversing gene silencing caused by CRABP2 methylation (Liu et al., 2019).

##### 3.1.1.2 RSV and histone modification

Most compounds that modulate epigenetics act through multiple mechanisms in cancer. Numerous studies have demonstrated that HDACs are frequently overexpressed in various cancer types, resulting in a notable decline in disease-free and overall survival. HDAC activity is a vital epigenetic target for ensuring survival and tumorigenesis. Notably, HDAC inhibitors are presently considered the most well-established epigenetic drugs developed to date.

In colon cancer, Resveratrol plays a multifaceted role involving mechanisms of DNA methylation and histone acetylation. The chemopreventive properties of Resveratrol require the involvement of the key player in epigenetic regulation, Sirtuin (SIRT) 1 constitutes a HDAC which oversees essential metabolic and physiological processes. Resveratrol stands out as one of the most effective activators of Sirt1, triggering the acetylation of substrates and NAD, thereby enhancing Sirt1 activity and leading to apoptotic cell death (Kolthur-Seetharam et al., 2006; Buhrmann et al., 2016). Abnormal methylation of the tumor suppressor genes p15 (INK)4(b) and p16(INK)4(a), along with the estrogen receptor-α (ESR1) gene, occurs in colon cancer. Resveratrol has been found to inhibit promoter methylation of p16(INK)4(a) and ESR1, which leads to inhibition of tumor cell growth (Berner et al., 2010).

##### 3.1.1.3 RSV and non-coding RNA regulation

Tertiary epigenetic regulation plays a role in modulating specific cancer events, primarily through post-transcriptional modifications of coding and non-coding RNAs that affect their function and homeostasis within the cell. Specific microRNAs (miRNAs) exhibit abnormal expression patterns in prostate cancer. The miRNA profile of prostate cancer is significantly modified by Resveratrol, comprising the oncogenic miR-17 family and miR-7 as well as the tumor-suppressive miR-296 and miR-654 (Kai et al., 2010; Kai et al., 2011; Kumar et al., 2015). Besides, overexpression of MTA1 in prostate cancer results in tumor invasion and metastasis. Resveratrol regulates histone acetylation, leading to the downregulation of MTA1 and subsequent MTA1/NuRD instability. This instability stimulates the acetylation and activation of downstream genes, namely, p53, NF-κB, and VEGF, representing another way in which Resveratrol indirectly impacts the epigenetic modification of downstream targets.

Breast cancer classification comprises estrogen-positive and triple-negative subtypes. In estrogen-induced breast cancer, Resveratrol acts as an inhibitor of Estradiol-mediated NRF2 promoter methylation and NRF2-targeted miR-93 expression (Singh et al., 2014). Triple-negative breast cancer is more aggressive clinically and unresponsive to traditional hormone therapy. Resveratrol induces significant downregulation of DNMT and HDAC in MCF-7 and MDA-MB-231 cells, leading to the restoration of BRCA1, p53, and p21 in cancer cells (Kala et al., 2015; Gao and Tollefsbol, 2018; Chatterjee et al., 2019).

#### 3.1.2 Resveratrol regulates metabolism

Furthermore, aside from its established effects on cancer treatment, Resveratrol has been shown to have pharmacological benefits for metabolic diseases, with studies indicating its positive influence on animals consuming a high-fat diet over extended periods. Resveratrol has been found to reduce high-fat diet-induced methylation of the Nrf2 promoter in mouse livers, leading to reduced triglyceride levels and lipogenesis. Nrf2 expression reduction has been associated with these effects in NAFLD (Hosseini et al., 2020). Resveratrol present in white adipose tissue (WAT) has the potential to regulate miRNA expression. Specifically, miR-539-5p plays a crucial role in inhibiting neoadipogenesis activated by Resveratrol in WAT. Such inhibition leads to a direct impact on treating obesity (Gracia et al., 2016).

#### 3.1.3 Resveratrol regulates nervous system

Additionally, Resveratrol has been reported to affect DNA methylation patterns in Parkinson’s disease, mainly by stimulating endogenous SIRT2, which results in CDKN2A DNA hypermethylation. This hypermethylation reduces the inhibitory impact of CDK4 and increases the expression of pRb, facilitating cell proliferation and growth (Li et al., 2021). As for anxiety disorders, pre-clinical studies suggest that Resveratrol’s modulatory effects on SIRTs have the potential to alleviate anxiety in pre-diabetic individuals (Reddy et al., 2016). In instances of lasting cerebral ischemia, Resveratrol has demonstrated effectiveness in diminishing brain damage and inflammation. It exhibits neuroprotective effects as a histone deacetylase inhibitor and SIRT 1 activator (Mota et al., 2020).

ε-Viniferin, a structurally-related compound to resveratrol, increased SIRT3 expression and FOXO3 deacetylation, decreased mitochondrial depolarization caused by rotenone, lessened neuronal apoptosis, and replenished mitochondrial homeostasis-associated proteins expression in a Parkinson’s model of SH-SY5Y cells following treatment (Zhang et al., 2020).

#### 3.1.4 Resveratrol regulates environmentally induced toxicity

Resveratrol also has a beneficial effect on regulating numerous physiological toxicities caused by environmental factors. Manganese and zinc may prompt apoptosis and abnormal epigenetic modification leading to reproductive toxicity, but Resveratrol counters these cytotoxic effects by preventing apoptosis and correcting any abnormal epigenetic modifications in cells (Wang et al., 2021). Neonatal exposure to general anesthetics and repeated administration of the same leads to neuropathological changes in the brain and cognitive deficits. In mice that were exposed to sevoflurane in the neonatal period, Resveratrol was found to enhance cognition and reverse SIRT1 expression (Tang et al., 2020).

These diverse examples underscore the pivotal role of Resveratrol in causing epigenetic changes associated with various diseases, offering intriguing prospects for targeted therapeutic interventions and further research into its epigenetic inheritance potential.

### 3.2 Trans-stilbenes: Pterostilbene

Pterostilbene is a methylated derivative of resveratrol, and its chemical formula is 3, 5-dimethoxy-4′-hydroxystilbene. It exhibits numerous biological activities, including antioxidant, anti-tumor, lipid-lowering, and anti-fungal activities. Compared to Resveratrol, Pterostilbene has a higher bioavailability. It plays a crucial role in epigenetic modifications linked to genetics and various diseases (Table 3).

**TABLE 3:** Epigenetic modulation of different diseases by Pterostilbene.

Name	Diseases type	Diseases	Regulated phenotypes	Regulatory mechanism	Stage
Pterostilbene	Cancer	Breast cancer	Mediated epigenetic inactivation of genes with oncogenic	DNA methylation	Preclinical
		Liver cancer	Inhibit cell proliferation and invasion	Histone acetylation	Preclinical
		Prostatic cancer	Inhibit cell proliferation	Histone acetylation	Preclinical
	Metabolism	High-fat diet obesity	Reduce adipose tissue gain	Histone methylation	Clinical
		Hyperlipemia	Inhibit PCSK9-induced hypercholesterolemia	MicroRNA Regulation	Clinical
		Diabetes	Accelerate diabetic wound healing	DNA methylation	Preclinical

#### 3.2.1 Pterostilbene regulates cancer

Pterostilbene has potent tumour-killing activity. In breast cancer, the heightened expression level of OCT1 is believed to play a role in both tumor formation and progression. Meanwhile, DNMT3B functions by adding methyl groups to oncogenes, leading to their silencing. Pterostilbene catalyzes the methylation of the OCT1 binding site through DNMT3B, leading to the failure of OCT1 to recognize its binding site and activate transcription, resulting in oncogene silencing. This suggests that it may act as an epigenetic modifier in the progression of breast cancer (Beetch et al., 2021). When combined with Resveratrol, Pterostilbene demonstrated a synergistic effect on TNBC cells, resulting in their efficient killing while remaining non-toxic to normal cells. This occurs as PTE can cause the reduction of SIRT1, restoration of histone acetylation, and normalization of expression of tumor suppressor genes (Kala et al., 2015; Beetch et al., 2019).

**FIGURE 3:**
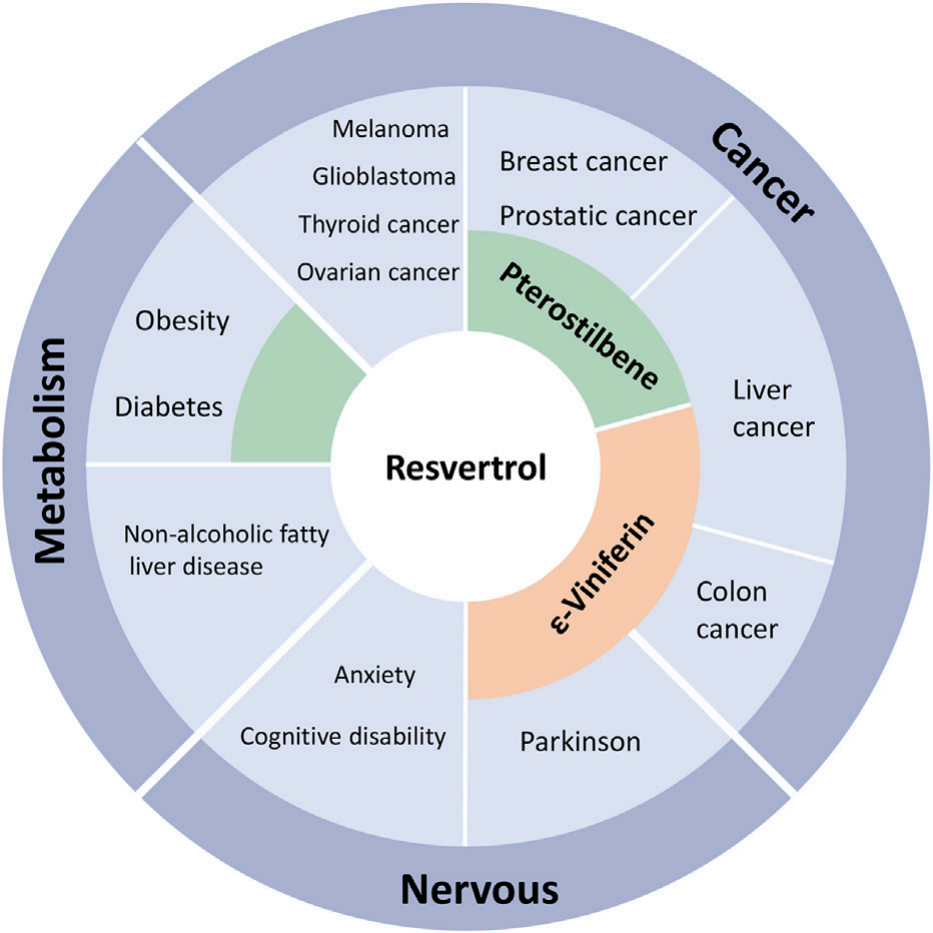
Association between Resveratrol and its derivatives, Pterostilbene and ε-Viniferin, on epigenetic regulation of different diseases.

As a structural derivative of Resveratrol, we can find that Pterostilbene and Resveratrol can modulate some common diseases (Figure 3). Similar to Resveratrol, Pterostilbene targets MTA1 in prostate cancer cells. MTA1 is responsible for deacetylating PTEN, an important tumor suppressor protein, thereby inhibiting its expression. Pterostilbene reduced MTA1-dependent cell survival and metastasis in prostate cancer by inhibiting MTA1 expression, disrupting the MTA1-histone deacetylase complex, and restoring PTEN expression (Dhar et al., 2016; Levenson, 2022a; Levenson, 2022b; Kocabas and Sanlier, 2023). In hepatocellular carcinoma, Pterostilbene decreased MTA1 expression and HDAC1 activity, disrupting the MTA1/histone deacetylase complex and leading to restored PTEN acetylation and subsequent induction of apoptosis in cancer cells (Qian et al., 2018). When combined with SAHA, a clinically approved HDAC inhibitor, Pterostilbene enhanced sensitivity to SAHA treatment by targeting MTA1 and HDAC via the same pathway in prostate cancer (Butt et al., 2017). These findings indicate that epigenetic alterations resulting from Pterostilbene can modify oncogenes via post-translational modifications, causing a significant impact on a range of cancer treatments. Notably, Pterostilbene exhibits the capacity to restrain HDAC activity and modify epigenetic mechanisms.

#### 3.2.2 Pterostilbene regulates metabolism

Additionally, Pterostilbene has the potential to counteract DNA methylation patterns induced by a high-fat diet and inhibit the upregulation of genes linked to obesity-related complications, such as fasn. Resveratrol was unsuccessful in altering the methylation pattern in the fasn gene region, whereas Pterostilbene was able to reverse the hypermethylation caused by the obesogenic diet at positions −90 bp and −62 bp (Gracia et al., 2014). In the context of hyperlipidemia, PCSK9 promotes the degradation of the low-density lipoprotein receptor (LDLR), resulting in hypercholesterolemia and myocardial dysfunction. Pterostilbene disrupts mRNA expression of important proteins, including PCSK35, by upregulating hsa-miR-9 and hsa-miR-2 expression, which impacts LDLR cholesterol mRNA expression and ultimately raises LDLR mRNA expression (Lin et al., 2021). Complications arising from diabetes significantly impact the quality of life in diabetic patients, as wound healing is impaired due to heightened inflammation and oxidative stress. As an antioxidant, Pterostilbene has ameliorated diabetes-induced epigenetic modifications of the ERβ promoter, as evidenced by a decrease in H3K9me2 and H3K27me3 modifications and an improvement in pro-inflammatory cytokine secretion in macrophages (Xie et al., 2021). These findings demonstrate the multifaceted role of Pterostilbenes in inducing epigenetic modifications linked to various diseases. They operate not only individually but also synergistically to enhance their effectiveness, providing interesting possibilities for focused therapeutic interventions and additional exploration of their epigenetic capabilities.

### 3.3 Cis-stilbene: Combretastatin A-4

Combretastatin A4 (CA4) is a potent inhibitor of the polymerization of microtubule proteins with strong suppressive effects on the growth of tumor cells. Notably, CA4 is structurally simple, easily modifiable, and has potent anti-angiogenic capacity (Paidakula et al., 2022). CA4 alters endothelial cell structure, disrupting vascular permeability and the tumor vascular system to inhibit angiogenesis (Sherbet, 2017). However, the clinical applications of stilbenes have been greatly limited due to their cis-structure, instability, poor water solubility, low bioavailability, and high toxicity (Young and Chaplin, 2004; Guo et al., 2022). CA4 has been identified as a promising lead compound for the development of antitumor drugs with improved stability. Many derivatives and analogs of CA4 have been developed to enhance water solubility and decrease adverse effects. Structural analogs of the CA4 nucleus were synthesized and found to be potent HDAC inhibitors with dual inhibition of HDAC and microtubules. The CA4 analog features a 1-(3-methoxyphenyl)-5-(3, 4, 5-trimethoxyphenyl)-1H-1, 2, 4-triazole-3-carboxamide structure (Aboeldahab et al., 2018).

### 3.4 Rigid compound: Raloxifene

Raloxifene is a drug that has received FDA approval and is used clinically to treat osteoporosis in postmenopausal women. Lysine-specific demethylase 1 (LSD1) has been identified as an epigenetic target in the treatment of the disease, and a small-compound library screen revealed that several LSD1 inhibitors, including Raloxifene, showed potential therapeutic effects by effectively inhibiting LSD1 activity (IC50 = 2.08 μM) and suppressing the proliferation and migration of renal cell carcinoma cells overexpressing LSD1 (Ma et al., 2021; Song et al., 2022). This indicates that Raloxifene may have therapeutic capabilities for RCC through the modulation of epigenetic elements.

In breast cancer research, estrogen exposure plays a crucial role in gene expression regulation. Researchers utilized Raloxifene, a selective estrogen receptor modulator, to investigate its impact on gene expression in breast cancer cells exposed to estrogen. The findings indicate that Raloxifene suppressed the expression of numerous estrogen-regulated genes, emphasizing its role in regulating estrogen-mediated gene expression (Englert et al., 2011). Furthermore, the study unveiled epigenetic regulatory components of particular genes, implying that Raloxifene may impact extended gene expression patterns in breast cancer cells.

In a separate investigation, scholars investigated the epigenetic control of the UDP-glucuronosyltransferase (UGT) 1A enzyme, which plays a role in the processing of different substances. It has been discovered that microRNA 491-3p (miR-491-3p) may act as a regulator for the UGT1A gene family, and that Raloxifene can influence the expression of the UGT1A1 gene. A decrease in miR-491-3p levels was found to suppress UGT1A1 expression and Raloxifene metabolism, whereas an increase was seen to have the opposite effect (Dluzen et al., 2014). This finding suggests that Raloxifene is involved in its own metabolic processes. Overall, Raloxifene appears to play a role in the epigenetic regulation of the UGT1A enzyme.

In conclusion, Raloxifene shows promise as an epigenetic modulator for disease treatment. It interacts with LSD1, impacts gene expression, and regulates epigenetic factors like miRNAs. These findings emphasize its significance in the field of epigenetics and its potential for regulating gene expression in diverse diseases.

## 4 Negative epigenetic regulation of Tamoxifen

### 4.1 Tamoxifen and breast cancer treatment resistance

Tamoxifen is the first medication approved by the FDA in the US for reducing the risk of breast cancer in high-risk women by up to 40%. Moreover, it is used to treat recurring or metastatic breast cancer, and as adjuvant therapy following surgery for early-stage breast cancer. Tamoxifen is an estrogen inhibitor that competes with estradiol for the estrogen receptor (ER) in the target organ. The Tamoxifen-ER complex affects gene transcription, thereby inhibiting tumor cell growth. Estrogen induces tumor cell proliferation and invasion in breast cancer (Osborne, 1998). Tamoxifen is employed as hormone therapy to manage ER-positive breast cancer patients. However, the therapeutic response of breast cancer cells to Tamoxifen may decline over time, resulting in drug resistance with an epigenetic connection (Badia et al., 2007; Abdel-Hafiz, 2017; Sukocheva et al., 2022). The mechanisms responsible for regulating epigenetic inheritance reside in four primary domains.

#### 4.1.1 Tamoxifen resistance and DNA methylation

Promoter hypermethylation regulates Tamoxifen resistance-related genes. The methylation of the ERα gene promoter region is often high in breast cancer cells that become Tamoxifen resistant. Such high methylation can inhibit the expression of the ERα gene which results in the reduction of ERα receptor levels, thereby decreasing the therapeutic efficacy of Tamoxifen. Prolonged use of Tamoxifen leads to changes in the activity of epigenetic enzymes EZH2 and DNMT, causing hypermethylation of the GREB1 promoter. This can result in Tamoxifen resistance, but development of EZH2 and DNMT inhibitors as therapeutic modalities could counteract this (Wu et al., 2018; Ye et al., 2019).

#### 4.1.2 Tamoxifen resistance and histone modification

Histone modification is another important epigenetic mechanism influencing Tamoxifen resistance. Histone modification patterns may change in drug-resistant breast cancer cells, such as histone acetylation and methylation. Treatment reversal with HDAC inhibitors re-established ER sensitivity to Tamoxifen (Raha et al., 2015). Furthermore, Tamoxifen could influence histone methylation status, especially H3K4 methylation and H3K9 methylation (Yu-Rice et al., 2016; Kim et al., 2020). Additionally, non-coding RNAs, such as microRNAs (miRNAs) and long non-coding RNAs (lncRNAs), can interfere with estrogen signaling pathways and ERα expression, potentially impacting Tamoxifen efficacy. For instance, lncRNA SNHG6 exhibits potential for modulating Tamoxifen resistance, while IncRNA SNHG101 enhances Tamoxifen sensitivity by suppressing miR-6 in Tamoxifen-resistant cells (Khan and Ahmad, 2022). Certain miRNAs can regulate resistance by targeted ERα or genes associated with Tamoxifen resistance. The miR-200 family is a renowned regulator of EMT, whereas c-MYB is involved in EMT induction. The c-MYB induction of EMT may lead to Tamoxifen resistance, but miR-200b and miR-200c can silence c-MYB resulting in the reversal of EMT and Tamoxifen resistance (Gao et al., 2019).

#### 4.1.3 Resveratrol and Pterostilbene may improve Tamoxifen resistance

Interestingly, both Resveratrol and Pterostilbene acted as inhibitors for DNMT and HDAC, resulting in the reactivation of ERα expression. Furthermore, there was an increase in the activities of Acetyl-H3, Acetyl-H3K9, and Acetyl-H4 in the ERα promoter region. Investigating the targets of Resveratrol and Pterostilbene that modulate epigenetic inheritance and those which contribute to Tamoxifen resistance enhanced the sensitivity of Tamoxifen for breast cancer treatment (Kala and Tollefsbol, 2016). Epigenetic modifications have a crucial function in regulating gene expression and chromatin structure, which can have a direct or indirect impact on the interactions between ERα and its target genes. Consequently, this alteration can alter the sensitivity of breast cancer cells to Tamoxifen.

### 4.2 Tamoxifen and breast cancer treatment recurrence

Not only does resistance develop following Tamoxifen treatment, but the likelihood of breast cancer recurrence also increases. About 50% of patients experience relapse during Tamoxifen therapy (Garcia-Martinez et al., 2021; Sher et al., 2022). The hypomethylation status of the NMT3A promoter and overexpression of DNMT3B may be implicated in disease recurrence among breast cancer patients treated with Tamoxifen. Promoter hypermethylation is also among the epigenetic regulators of Tamoxifen resistance (Hilakivi-Clarke et al., 2017; Jahangiri et al., 2018).

### 4.3 Tamoxifen treatment leads to other diseases

In addition to Tamoxifen’s therapeutic and resistance mechanisms related to epigenetic regulation, certain studies have indicated that Tamoxifen use elevates the risk of other diseases, including endometrial cancer and liver cancer. Furthermore, patients receiving Tamoxifen treatment tend to develop high-grade tumors and have a diminished prognosis. The process of promoter hypermethylation of the DNA repair enzymes MGMT and CXCL12 results in heightened methylation of the CpG islands of the respective promoters since they control gene transcription. The modification of DNA methylation processes contributes to how Tamoxifen induces endometrial cancer (Kubarek and Jagodzinski, 2007; Nagy et al., 2014). Tamoxifen is an anti-estrogen prescribed for treating breast cancer, it also accelerates hepatocellular carcinoma development and progression through mechanisms associated with epigenetic regulation. In liver tissue, chronic Tamoxifen exposure in rats led to decreased DNA methylation and histone H4K20 trimethylation. These epigenetic modifications solely occurred in the liver and did not affect other tissues like the pancreas, spleen, and mammary gland (Tryndyak et al., 2006).

## 5 Clinical trial

### 5.1 Status of clinical trial

**FIGURE 4:**
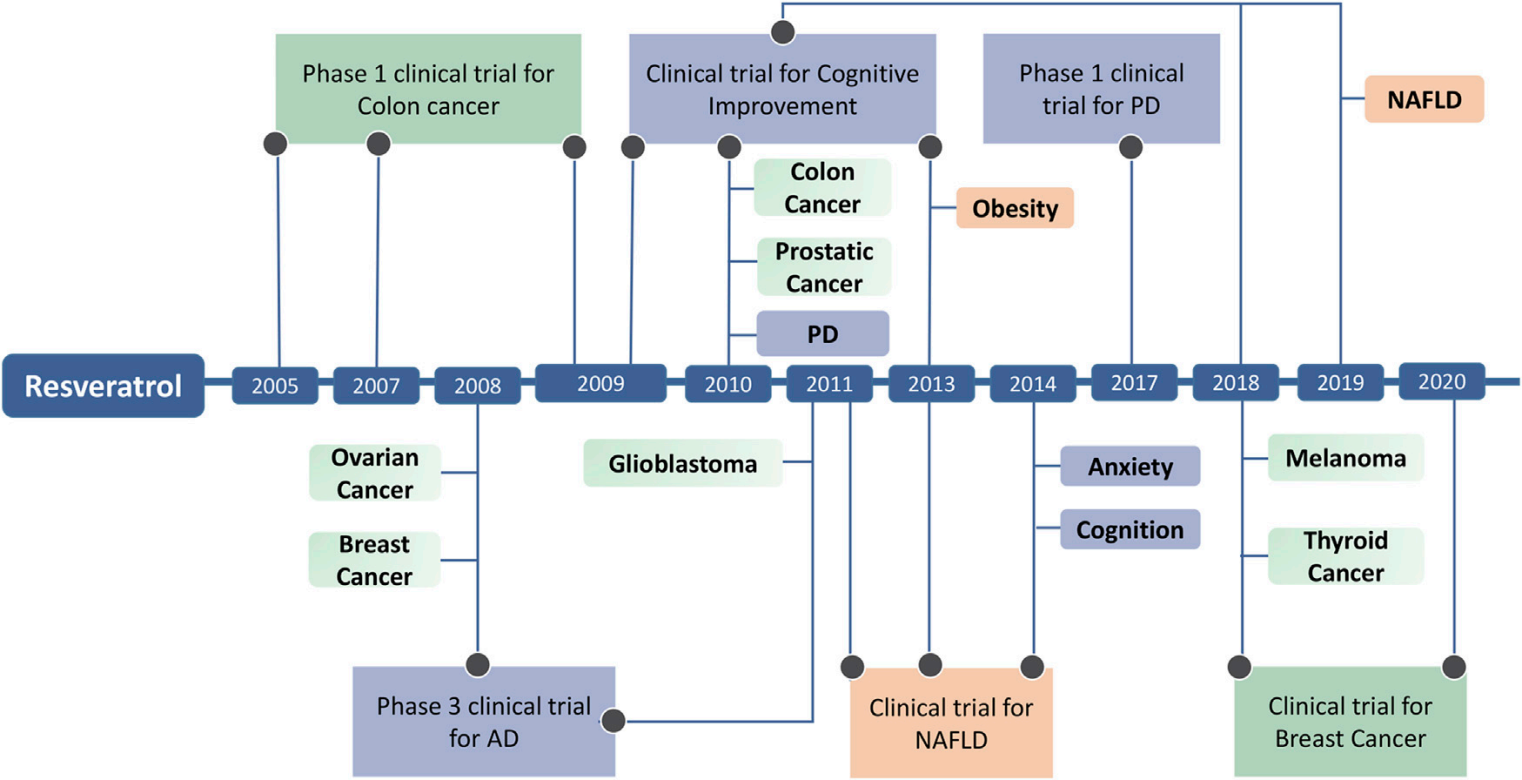
The progression of Resveratrol modulates disease through epigenetic regulation and Clinical Trials of Resveratrol.

Stilbenes have shown promise in clinical applications for diseases with epigenetic components (Table 4). Notably, numerous clinical trials have explored the potential epigenetic effects of Resveratrol (Figure 4). In two completed Phase I trials (NCT00920803 and NCT00433576), researchers investigated the safety and pharmacodynamics of Resveratrol in patients with colon cancer, and another Phase I clinical trial (NCT00256334) tested whether Resveratrol, which develops in colon cancer and normal colon mucosa, regulates Wnt signaling *in vivo*, finding that Resveratrol could prevent colon cancer (Nguyen et al., 2009; Howells et al., 2011). Breast cancer is characterized by mutations in tumor suppressor genes such as BRCA1 and BRCA2, which increase the risk of breast and ovarian cancer in carriers. In this pathology, DNA damage is increased due to the presence of a chronic inflammatory state coupled with oxidative stress caused by anti-tumor therapy and changes in body composition. By inducing epigenetic changes through Resveratrol intervention, trial NCT05306002 assessed whether DNA damage was reduced in patients after the intervention. Non-alcoholic fatty liver disease (NAFLD) and fatty liver (NASH) patients are increasing annually and are strongly associated with obesity. There are no known effective treatments. From animal studies, it is known that the compound Resveratrol may have the potential to neutralize obesity-induced diseases. Four clinical trials focused on the effects of Resveratrol on fatty liver disease in overweight adolescents and men. Trial NCT02244879 assessed whether increased SIRT-1 expression affects the acetylation of histone 3 at lysine residue 56 (H3K56ac) in patients, with results suggesting that promoting SIRT-1 activation affects redox homeostasis in these patients (Bo et al., 2018).

**TABLE 4:** Clinical trials of stilbenes in clinical stage.

NCT number	Drug	Dose/day	Disease	Phase	Status
NCT00433576	Resveratrol	—	Colon cancer	Phase 1	Completed
NCT00920803		5,000 mg	Colon cancer	Phase 1	Completed
NCT00256334		20–160 mg	Colon cancer	Phase 1	Completed
NCT03482401		474 mg	Breast cancer	Not applicable	Completed
NCT05306002		—	Breast cancer	Not applicable	Active, Not recruiting
NCT01464801		500 mg	NAFLD	Not applicable	Completed
NCT02030977		500 mg	NAFLD	Phase 2 and 3	Completed
NCT01446276		1,500 mg	NAFLD	Not applicable	Completed
NCT02216552		150 mg	NAFLD	Phase 2 and 3	Completed
NCT03095092		400 mg	PD	Phase 1	Completed
NCT03093389		25–150 mg	PD	Phase 1	Completed
NCT03095105		200 mg	PD	Phase 1	Completed
NCT03094156		25–100 mg	PD	Phase 1	Completed
NCT01794351		500 mg	Cognitive improvement	Not applicable	Completed
NCT03546075		500 mg	Cognitive improvement	Not applicable	Completed
NCT04314739		500 mg	Cognitive improvement	Not applicable	Completed
NCT01010009		500 mg	Cognitive improvement	Not applicable	Completed
NCT03448094		500 mg	Cognitive improvement	Not applicable	Completed
NCT01219244		—	Cognitive improvement	Phase 2 and 3	Completed
NCT01504854		1,000 mg	AD	Phase 2	Completed
NCT00678431		5 mg	AD	Phase 3	Completed
NCT05561075	Pterostilbene	—	Bioavailability	Not applicable	Completed
NCT01267227		100–250 mg	Cholesterol, Antihypertensive	Phase 2 and 3	Completed
NCT03671811		—	Endometrial cancer	Phase 2	Completed
NCT04562831		200–300 mg	ALS	Not applicable	Recruiting
NCT04530916		—	Vascular-Protective	Phase 1 and 2	Recruiting
NCT00060242	Combretastatin A-4	—	Thyroid cancer	Phase 2	Completed
NCT00003768		—	Advanced solid tumors	Phase 1	Completed
NCT00003698			Solid tumors	Phase 1	Completed

These trials collectively shed light on the potential of Resveratrol to influence epigenetic processes in different disease contexts, particularly cancer and aging-related conditions. While the specific outcomes and implications of these trials may vary, they contribute to our growing understanding of how Resveratrol, as an epigenetic modulator, may play a role in disease prevention and treatment. Ongoing research in this area may provide further insights into the clinical applications of Resveratrol and its impact on epigenetics, potentially opening new avenues for personalized therapies and anti-aging interventions. However, several challenges and opportunities lie ahead. One challenge is optimizing the dosage and bioavailability of stilbenes, as their effective concentrations may vary among individuals and disease contexts. Moreover, the intricate interplay between different epigenetic marks requires further research. Understanding the context-dependent nature of stilbenes effects is crucial to accurately tailor treatments for specific diseases.

### 5.2 Challenge for clinical trial

Researchers are actively exploring personalized epigenetic therapies, with the aim of targeting specific epigenetic alterations unique to individual patients. Another avenue of investigation involves the design of novel stilbene derivatives with improved specificity and potency. These derivatives have the potential to offer enhanced epigenetic regulation while minimizing off-target effects. Innovative drug delivery systems, such as nanoparticles and liposomal formulations, are being explored to enhance the bioavailability of stilbenes and enable tissue-specific targeting (Zhang et al., 2022).

Nonetheless, several questions remain unanswered. These include concerns regarding the long-term safety of stilbene-based treatments, potential resistance mechanisms, and the precise molecular pathways through which stilbenes exert their epigenetic effects in different disease contexts. These challenges underscore the complexity of epigenetic regulation in disease and emphasize the necessity for ongoing research and innovation.

Furthermore, critical questions persist. It is imperative to gain a deeper understanding of the exact mechanisms through which stilbenes modulate epigenetic marks in different diseases. Additionally, the long-term effects of stilbene-based treatments on epigenetic stability and the possibility of epigenetic rebound after treatment cessation need thorough investigation. The development of standardized protocols for stilbene-based epigenetic therapies, encompassing dosing, duration, and patient selection criteria, is also a matter of pressing concern.

In conclusion, stilbenes hold tremendous potential as epigenetic modulators in disease management. Current clinical applications, particularly in the domains of cancer, cardiovascular diseases, and neuroprotection, have yielded promising results. However, challenges such as dosage optimization and safety considerations must be diligently addressed. Future developments in personalized therapies, novel derivatives, and drug delivery systems offer exciting prospects. Emerging research areas, including combination therapies and biomarker identification, hold the promise of advancing the treatment of epigenetic diseases. Nevertheless, many questions remain, underscoring the necessity for ongoing research to fully harness the therapeutic potential of stilbenes in the intricate realm of epigenetics.

## 6 Conclusion and prospect

**FIGURE 5:**
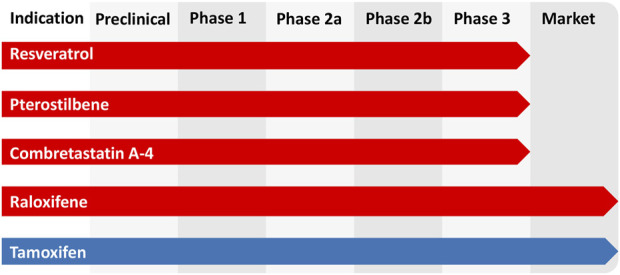
Clinical study phase of stilbenes.

The role of the parent compound is pivotal in the realm of small molecule drug discovery and development, as it serves as the cornerstone for the synthesis of targeted compounds. Pterostibene compounds, including Resveratrol and Pterostibene, hold substantial significance in the field of epigenetic regulation. Their potential to influence the epigenetic landscape and facilitate disease management has been well-documented. These compounds exhibit unique structural characteristics that modify critical epigenetic markers, specifically DNA methylation and histone modifications. Functionally, stilbenes can induce favorable or unfavorable epigenetic modifications in various diseases. Stilbenes can positively modulate disease through epigenetic mechanisms by reshaping gene expression patterns, thereby exerting a beneficial influence on diverse diseases, including cancer, cardiovascular disease, and neurological disorders. Furthermore, all of these stilbenes have reached the clinical research stage, with some already available as antitumor medications (Figure 5), underscoring their developmental potential. Consequently, conducting comprehensive research on the biological activities, regulatory mechanisms, and derivatives of stilbenes is indeed worthwhile. As epigenetic research continues to progress, new areas of exploration are emerging. One promising avenue involves the development of epigenetic combination therapies that integrate stilbenes with other epigenetic modifiers, chemotherapeutic agents, or immunotherapies. These synergistic approaches hold the potential to offer more comprehensive and effective disease management strategies. Additionally, there is a growing interest in identifying biomarkers that can predict patient responses to stilbene-based epigenetic therapies, facilitating the customization of treatment plans.

However, it’s worth noting that only a minority of stilbenes induce adverse effects via epigenetic regulation. Moreover, we have uncovered that stilbenes not only provide treatment for specific illnesses through epigenetic processes but also play a role in the resistance mechanisms of certain drugs, potentially contributing to the recurrence and development of particular conditions. These findings contribute to a deeper understanding of the negative epigenetic regulatory mechanisms of these substances. Improving the comprehension of adverse drug reactions and toxic side effects is integral to enhancing patient care. It is widely acknowledged that compounds can specifically bind to their targets, offering an opportunity to tailor small molecules to mitigate side effects by exploring epigenetic regulatory mechanisms specific to target sites, and subsequently modifying compound structures.

Stilbenes represent a promising therapeutic option for epigenetically driven disease treatment. Their potential for personalized therapeutic approaches and versatility in targeting diverse diseases make this class of compounds highly promising for treatment. It necessitates in-depth investigation and development. However, careful attention must also be given to challenges such as optimizing dosages for clinical trials and addressing long-term safety concerns. As we navigate the evolving landscape of epigenetic research and therapeutic development, ongoing investigations into stilbenes, their derivatives, and innovative drug delivery systems will be of paramount importance. These efforts will ultimately shape the future of stilbene-based epigenetic therapies, providing new strategies to combat epigenetically regulated diseases and improve patient outcomes.
